# Regional gray matter volume is associated with trait modesty: Evidence from voxel-based morphometry

**DOI:** 10.1038/s41598-017-15098-x

**Published:** 2017-11-02

**Authors:** Chuhua Zheng, Qiong Wu, Yan Jin, Yanhong Wu

**Affiliations:** 1 School of Psychological and Cognitive Sciences, Peking University, Beijing, China; 2 McGovern Institute for Brain Research, Peking University, Beijing, China; 3 Beijing Key Laboratory of Behavior and Mental Health, Peking University, Beijing, China

## Abstract

Modesty when defined as a personality trait, is highly beneficial to interpersonal relationship, group performance, and mental health. However, the potential neural underpinnings of trait modesty remain poorly understood. In the current study, we used voxel-based morphometry (VBM) to investigate the structural neural basis of trait modesty in Chinese college students. VBM results showed that higher trait modesty score was associated with lager regional gray matter volume in the dorsomedial prefrontal cortex, right dorsolateral prefrontal cortex, left superior temporal gyrus/left temporal pole, and right posterior insular cortex. These results suggest that individual differences in trait modesty are linked to brain regions associated with self-evaluation, self-regulation, and social cognition. The results remained robust after controlling the confounding factor of global self-esteem, suggesting unique structural correlates of trait modesty. These findings provide evidence for the structural neural basis of individual differences in trait modesty.

## Introduction

Modesty, often defined as the “public under-representation of one’s favorable traits and abilities (p. 473)^1^”, has recently garnered the attention of psychologists, particularly with the resurgence of interest in positive psychology and character virtues^2,3^. Some researchers have considered modesty as a behavioral self-presentation, used in order to obtain positive social images and results^4,5^. While, other researchers have argued that modesty can also be defined as a personality disposition, a trait that remains consistent across time and situations^6^. As an attribute of personality, modesty or trait modesty has been increasingly recognized as an important component in personality structures such as the Big Five^1^ and the HEXACO model^7^ (which includes six basic factors: Honesty-Humility, Emotionality, Extraversion, Agreeableness, Conscientiousness, and Openness to Experience). Trait modesty reflects personal thoughts, feelings and actions about themselves in comparison to other people^6^, indicating how an individual appraises himself/herself. People with high trait modesty scores tend to be unassuming and view themselves as ordinary people without any claim to special treatment, whereas low scorers tend to be self-enhancing and consider themselves as superior and entitled to special privileges^8^. Previous studies have indicated that trait modesty is a reliable predictor of individual differences in diverse self-reported criteria and behaviors^9^. Trait modesty has been associated with positive life outcomes, such as stable interpersonal relationship^10^, positive social evaluation^11^, and better group performance^12^. Furthermore, evidence indicates that trait modesty is beneficial to adaptive psychological functioning. For example, people with high trait modesty scores are less angry, hostile, and aggressive towards others and show better social adjustments^13,14^. Trait modesty is also related to a greater sense of psychological well-being^2^, and entails serving a valuable social function by reducing conflict and resentment^15^. These studies suggest that trait modesty is a valuable disposition with significant practical functions in crucial domains such as the workplace, interpersonal and social function, and mental health. Despite the numerous studies focused on modesty and its beneficial effects for multiple life outcomes, the neural correlates of trait modesty remain unclear.

By explaining the psychological processes involved in trait modesty, it is beneficial in further understanding the neural mechanisms of trait modesty. Personality and social psychological research has suggested that trait modesty involves a cognitive, self-regulatory and social component^14^, which are formed by self-evaluation, self-regulation and social cognition, respectively. When considering the cognitive component of trait modesty, trait modesty has been regarded as a form of psychological self-evaluation, with Sedikides *et al*.^14^ defining it as “*a moderate self-view* [italics in original]”. It involves the perception of one’s ability or achievement and is linked to self-reflection and cognitive evaluation process. Moreover, trait modesty provides modest individuals with accurate perceptions of their abilities and ample self-esteem, which facilitates self-regulatory abilities to resist self-enhancing tendencies and to generate adaptive consequences^14^. Previous research has indicated that modest individuals are able to regulate egotism in socially appropriate ways^14^, so they may achieve social acceptance and maintain interpersonal harmony, especially in collectivist cultures^16,17^. Compared to self-enhancing people, modest people are more prudent risk-takers^18^ and are more focused on long-term objectives rather than fulfilling short-term emotional needs (e.g., feeling good about themselves)^19^. In addition, modesty is often manifested in social interactions, involving a comparison between the self and others^16^. Trait modesty delineates an individual’s tendency to care for others and concern about relationships with others in social comparison. For example, downplaying personal achievements after one’s success, in order to reduce the threat to others’ self-esteem, while also elevating others through expressions of gratitude and appreciation^17^. Trait modesty is associated with prosociality and relational harmony^14,17^, and has also been characterized by traits such as solicitousness, plainness, helpfulness, empathy, agreeableness, gratitude, and forgiveness^20,21^. These aspects reflect a social component of trait modesty, involving in the perception of other’s needs and feelings and the social situations. In summary, these studies suggest that trait modesty is linked to psychological processes such as self-evaluation, self-regulation, and social cognition.

Although there has been no direct evidence of neural correlates of modesty, previous neuroimaging studies have provided insights into the potential brain mechanisms underlying trait modesty. Previous research has consistently revealed the involvement of the cortical midline structures (CMS) in self-referential processing^22–24^. The CMS consists of the dorsomedial prefrontal cortex (DMPFC), ventromedial prefrontal cortex, anterior cingulate cortex and posterior cingulate cortex^22^. The DMPFC is activated during self-evaluation and general self-reflection^24–26^. Other studies have revealed that several areas of the prefrontal cortex (PFC), such as the dorsolateral prefrontal cortex (DLPFC), the ventrolateral prefrontal cortex, and the ventromedial prefrontal cortex, play a role in cognitive control^27^, emotion regulation^28^, self-control^29^ and self-regulation^30^. Furthermore, alterations in these regions were found to lead to dysregulation of social behavior, abnormal emotional expression, and cognitive deficits^31,32^. Given that trait modesty has been suggested as a cognitive self-evaluation and self-regulatory process, we speculated that certain regions of the PFC, especially the DMPFC and the DLPFC may be associated with trait modesty.

In addition, trait modesty has been linked to the tendency to focus on the concerns of other people, which involves in the process of dealing with social information (e.g., perceiving, thinking about, and making sense of oneself and others in the social world)^33,34^. This process is associated with brain structures related to the social cognition network. Previous research has shown that the involvement of the superior temporal gyrus (STG), temporo-parietal junction (TPJ), temporal pole (TP), posterior cingulate cortex (PCC)/precuneus, medial and lateral frontal regions are important in guiding social behaviors in this social network^33–35^. Moreover, recent structural magnetic resonance imaging (MRI) studies have demonstrated that regional variation in brain morphemetry (e.g., gray matter volume and cortical thickness) is correlated with individual differences in behavior, cognition, and more importantly, personality traits^36–40^. For example, DeYoung *et al*.^41^ found that the different dimensions of Big Five personality traits were associated with regional differences in gray matter volume (GMV) in specific brain regions. Agreeableness, which is closely and positively associated with trait modesty^6,42^, was also associated with reduced volume in the posterior left superior temporal sulcus and with increased volume in the PCC, both being areas that are involved in the processing of social information^43^. These findings suggest that the social cognition network may be engaged in the formation of trait modesty.

Based on previous behavioral and brain imaging studies, we hypothesized that individual differences in trait modesty would be associated with regional GMV (rGMV) in regions involving in self-evaluation, self-regulation, and social cognition, including the PFC (e.g., DMPFC, DLPFC) and social cognition network (e.g., STG, TPJ). In this study, structural magnetic resonance images were acquired from Chinese college students, and trait modesty was measured by the Honesty-Humility Modesty facet scale of the Chinese version of HEXACO Personality Inventory-Revised (HEXACO-PI-R)^44^. To test our hypotheses, we used voxel-based morphometry (VBM)^45^ to examine the associations between individual differences in trait modesty and brain structure differences in rGMV.

## Results

### Trait modesty score

Table 1 showed the means, standard deviation (SD), skewness, and kurtosis for trait modesty and self-esteem score. The kurtosis and skewness of trait modesty and self-esteem score were within range between −1 and +1, confirming the normality of the data^46^. One sample *t*-test comparing the midpoint of the trait modesty scale revealed that the current sample had a high level of trait modesty [*t* (49) = 5.07, *p* < 0.001], which is in line with the results of previous studies that modesty is highly valued in Chinese culture^17,47^. In addition, the trait modesty score was not significantly correlated with age (*r* = −0.03, *p* = 0.842), global GMV (*r* = −0.04, *p* = 0.793), and self-esteem (*r* = −0.04, *p* = 0.793). No significant gender difference in trait modesty [*t* (48) = −0.54, *p* = 0.594] was found.

**Table 1:** Descriptive statistics for self-esteem and trait modesty scores (n = 50).

	Means (SD)	Range	Skewness	Kurtosis
Trait modesty	27.00 ± 4.18	14–36	−0.34	0.83
Self-esteem	32.70 ± 4.22	23–40	−0.10	−0.67

### Correlation between rGMV and trait modesty score

**Figure 1:**
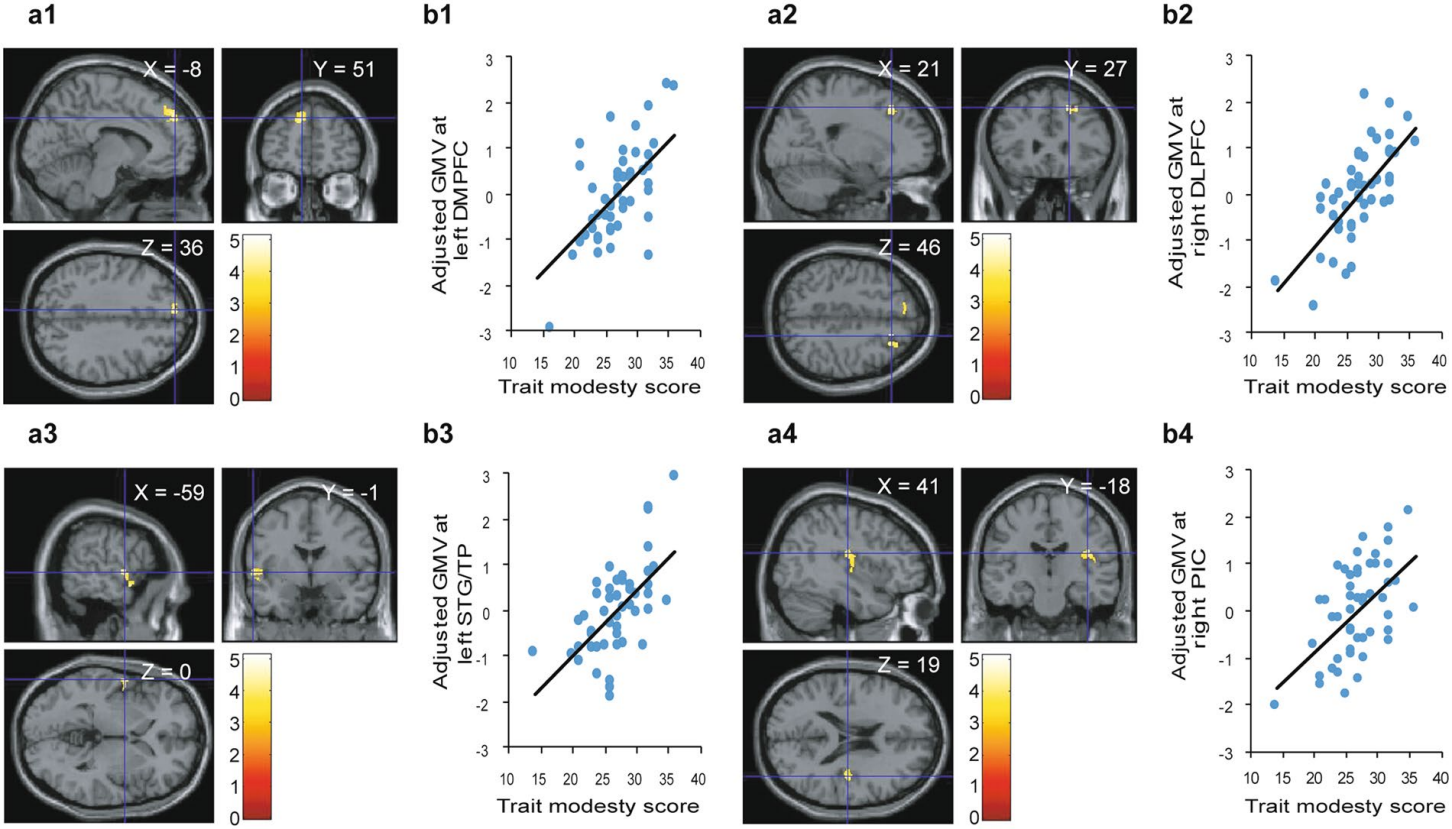
Regions showing associations between regional gray matter volume (rGMV) and trait modesty score. The red-to-yellow color scale indicates the t-score for the positive association between rGMV and trait modesty score (*p*_*FWE*_ < 0.05 at cluster level with an underlying voxel level of *p*_*uncorr*_ < 0.001; k > 200 for visualization purposes). Regions showing associations were overlaid on single T1 image in SPM8 toolbox. Areas of significant associations are shown in (**a1**) the left dorsomedial prefrontal cortex (DMPFC), (**a2**) the right dorsolateral prefrontal cortex (DLPFC), (**a3**) the left superior temporal gyrus (STG)/left temporal pole (TP) regions, and (**a4**) the right posterior insular cortex (PIC). Scatterplot of the correlation between the trait modesty score and mean rGMV values in the significant clusters in (**b1**) the left DMPFC, (**b2**) right DLPFC, (**b3**) left STG/TP, and (**b4**) right PIC are shown.

We investigated rGMV associated with individual differences in trait modesty. VBM results showed a significant positive correlation between the trait modesty score and rGMV in the left STG/left TP (peak voxel of MNI: x = −59, y = −1, z = 0, *Z* = 4.53, *p*_*FWE*_ < 0.001 at cluster level), left DMPFC (peak voxel of MNI: x = −8, y = 51, z = 36, *Z* = 4.04, *p*_*FWE*_ = 0.008 at cluster level), right posterior insular cortex (PIC) (peak voxel of MNI: x = 41, y = −18, z = 19, *Z* = 3.90, *p*_*FWE*_ = 0.016 at cluster level), and a (marginal) positive association with right DLPFC (peak voxel of MNI: x = 21, y = 27, z = 46, *Z* = 4.50, *p*_*FWE*_ = 0.077 at cluster level) after controlling for age, gender and global GMV (See Fig. 1, Table 2). No negative correlations between trait modesty and rGMV were observed.

**Table 2:** Regional gray matter volume (rGMV) significantly correlated with trait modesty.

Regions	Laterality	BA	Cluster		Peak			
			*k*	*Volume* (mm^3^)	x	y	z	*Z*-value
STG/TP	L	22/38	566	1910 mm^3^	−59	−1	0	4.53
DLPFC	R	8/9	214	722 mm^3^	21	27	46	4.50
DMPFC	L	6/8/9	357	1205 mm^3^	−8	51	36	4.04
PIC	R		313	1056 mm^3^	41	−18	19	3.90

Note: L = left; R = right; STG = superior temporal gyrus; TP = temporal pole; DLPFC = dorsolateral prefrontal cortex; DMPFC = dorsomedial prefrontal cortex; PIC = posterior insular cortex. Statistical maps were thresholded at *p*_*uncorr*_ < 0.001; all clusters were *p*_*FWE*_ < 0.05 at cluster level.

To examine whether these results were specific to trait modesty, we also excluded a confounding factor of global self-esteem. Another multiple regression model was conducted with global self-esteem included as covariate in addition to age, gender, and global GMV. Although there were small variations in cluster size, all correlations remained significant after age, gender, global GMV, and global self-esteem had been controlled (left STG/left TP: peak voxel of MNI: x = −56, y = 11, z = −12, *Z* = 4.45, *p*_*FWE*_ = 0.001 at cluster level; left DMPFC: peak voxel of MNI: x = −8, y = 51, z = 36, *Z* = 4.01, *p*_*FWE*_ = 0.013 at cluster level; right PIC: peak voxel of MNI: x = 41, y = −18, z = 19, *Z* = 3.82, *p*_*FWE*_ = 0.029 at cluster level), and a (marginal) positive association with right DLPFC (peak voxel of MNI: x = 21, y = 27, z = 46, *Z* = 4.41, *p*_*FWE*_ = 0.099 at cluster level).

## Discussion

The present study provides direct evidence regarding the brain structures underlying individual differences in trait modesty. The results of our analysis revealed that trait modesty score was positively correlated with rGMV in the left DMPFC, right DLPFC, left STG/TP, and right PIC, suggesting that neural pathways underlying self-evaluation, self-regulation, and social cognition are associated with trait modesty.

First, we found a positive association between rGMV in the DMPFC and trait modesty, which is consistent with the results of previous research documenting the role of the DMPFC in the processes of reappraisal and evaluation of self-relevant information^22,48^. For example, Kelley *et al*.^23^ and Johnson *et al*.^26^ found an increased DMPFC activation in the processes of self-referential evaluation regarding personal traits and abilities, while Fossati *et al*.^25^ confirmed that the DMPFC is important for the self-referential processing irrespective of the valence of processed personality traits. Moreover, Wu *et al*.^49^ observed that positive self-evaluation was associated with increased resting-state function activity in the DMPFC (including the DLPFC), indicating that these brain regions play a key role in maintaining spontaneous positive self-evaluative tendencies. Consequently, we can speculate that being modest may function as a strategy for maintaining positive self-evaluations among the Chinese. Modest Chinese participants will manifest lower explicit self-evaluation while manifesting higher implicit self-evaluation^48^. In other words, trait modesty can be perceived through outward self-effacing presentations such as not bragging or downplaying personal achievements, but not necessarily a lack of self-confidence or self-esteem^6,17^ This is consistent with previous research, which demonstrated that Chinese modesty is not just a form of self-effacing presentations but also functions as self-enhancement as well^16,47^. Furthermore, the DMPFC is also critical to social interactions, such as s comparing oneself to others^24^. As modesty is often manifested in social comparison, this result may indicate that modest individuals process information about the self in relation to others rather than context-independent self-views.

Additionally, the DLPFC is considered to play a critical role in the cognitive control^27^ and emotion regulation processes^28^. This region is also found to be involved in self-regulation, such as regulating goal-directed interpersonal behavior to adapt to social norms (e.g., altruism and fairness norms)^50–52^. Based on this, even though there was only marginal significance, increased rGMV in the DLPFC linked to trait modesty is consistent with more modest individuals having increased self-regulatory abilities^14,18,19^. Increased self-regulation might enable a modest person to regulate his/her behaviors in social interactions^14,19^, which is important for promoting better social adjustment and psychological health. Therefore, the results of our study suggest that those with higher tendency for trait modesty have increased self-regulation, thus further contributing to elicit a range of positive outcomes.

We further observed that trait modesty was correlated with brain regions that have been demonstrated to be engaged in complex social functions required for successful social interactions. Individual differences in trait modesty were positively associated with rGMV in the STG and TP, which have an important role in social cognition relating to the perception and comprehension of others’ mental states, feelings, thoughts, enduring dispositions, and actions^33,34^. These associations are consistent with the hypothesis that trait modesty is associated with the social information processing and interpersonal perception that enables and motivates prosocial behavior. Specifically, previous studies have shown that the STG serves an important role in social emotional processing^53,54^, the representation of abstract social concepts/values, moral sentiments^55^, and moral cognition^56^. Recent studies have suggested that the TP is a convergence zone of social information, where the brain organizes and defines the information into social scripts, a process important for mentalizing^57,58^. The processes of specifying the kinds of thoughts, feelings and behaviors that are most likely to occur in a particular context enable us to consider the perspectives of others. These functions may directly relate to modesty, as modesty has been viewed as social scripts used to guide appropriate, correct or desirable behaviors for social interactions in Chinese society^16^. Previous studies have suggested that individuals with high trait modesty are more interpersonally robust, more concerned about others’ needs and feelings, and are better able to take the perspectives of others^14,20,59^. Therefore, in this study, the positive relationship between trait modesty and rGMV in the STG and TP may indicate an increased ability of social cognition among modest individuals.

In addition, we found that trait modesty was correlated with rGMV in the right PIC. The PIC was found to be involved in the processing of fairness related moral judgment^60^. In accordance with this finding, scholars have claimed that modesty is related to justice and egalitarianism because modesty allows people to have an accurate perception of self and others^15^. Furthermore, previous research has shown that modest individuals were fair and genuine in dealing with others in the economic decision-making tasks^61^. Thus, it is plausible that modest people’s appreciations for justice and egalitarianism result in increased rGMV in the PIC.

There are some limitations in our study. First, type II error (false negatives) cannot be ruled out. This suggests that there may be further regions that are volumetrically related to trait modesty, but were rejected due to insufficient statistical power, and future research might address this possibility. However, our sample size has been considered appropriate for whole-brain analyses^62,63^. Furthermore, given that findings remained significant after a family-wise error corrected for multiple comparisons as done in prior VBM studies, our findings still make a meaningful contribution to our understanding of trait modesty. Second, the analyses of the current study are correlational in nature, and a longitudinal design is needed to determine the causal direction between modest trait and changes in brain structure. It should be noted that modesty is influenced by different regions and culture^12,64^ and our sample only consists of Chinese participants. As collectivism and social interdependence are an integral part of Chinese culture^12^, the results of our study might only be representative of individuals who grew up in a collectivist culture or hold interdependent self-construal. Future research should examine participants influenced by individualistic cultures to explore the neural basis underlying individual differences in trait modesty. Finally, previous studies suggested that there were two elements of trait modesty, intrapersonal (e.g., self-evaluation) and interpersonal (e.g., social orientation)^14,20^. However, this study is only an exploratory structure analysis and did not examined these two elements of trait modesty separately due to the lack of an established measure. In the future, as the next step to further analysis, we hope to develop a highly reliable measure that assesses these two dimensions of trait modesty and to explore whether the different dimensions of trait modesty are associated with gray matter differences.

In conclusion, the present study successfully identified potential neural correlates of trait modesty using VBM approach. We found that individual differences in trait modesty are linked to brain regions associated with self-evaluation processing, self-regulation, and social cognition. Moreover, these findings were maintained after controlling for individual differences in global self-esteem, suggesting a unique structural basis for individual differences in trait modesty. The results of the present study are the first step in exploring the neural correlates of trait modesty and will begin the advancement of our understanding of the nature and function of modesty.

## Method

### Participants

50 healthy adult volunteers (25 males, and 25 females, mean age ± standard deviation = 22.22 ± 2.37 years, age range: 18–29 years) were recruited from Peking University. All participants were right-hand (except for one participant) and none of them reported a history of neurological or psychiatry disease, or substance abuse. Written informed consent was obtained from each participant. This study was approved by and conducted in accordance with the Human Subjects Review Committee of Peking University.

### Assessment of trait modesty

The current study used the Honesty-Humility Modesty facet scale of the Chinese 200-item version of the HEXACO Personality Inventory-Revised (HEXACO-PI-R)^44^ to assess participants’ levels of trait modesty. The HEXACO trait modesty scale consists of eight items measuring individual differences in their tendency towards being modest and unassuming; for example, “I am an ordinary person who is no better than others”, “I would not want people to treat me as though I were superior to them”, “I am special and superior in many ways” (reverse coded), and “I think that I am entitled to more respect than the average person is” (reverse coded). High scores suggest that people see themselves as ordinary people without any claim to special treatment, whereas low scores consider themselves as superior to other people and feel entitled to special privileges^8^. Ratings are made on a 5-point Likert-type scale from 1 (strongly disagree) to 5 (strongly agree). The HEXACO trait modesty scale was found to have a higher internal consistency, Cronbach’s coefficient of α = 0.83^8^. For more information on the inventory, the scales, and all items, see hexaco.org.

### Assessment of self-esteem

The Rosenberg Self-esteem Scale (RSES)^65^ is a 10-item self-report questionnaire measuring global self-esteem. Example items are “I am able to do things as well as most other people” and “I feel I do not have much to be proud of” (reverse coded). All items are rated on a 4-point Likert-type scale ranging from 1 (strongly disagree) to 4 (strongly agree), Higher scores indicates higher self-esteem. The RSES has been widely used in many countries^66^. The Chinese version of this scale has been demonstrated to be reliable and valid in assessing self-esteem in Chinese populations^67^. In this study, the internal consistency (Cronbach α) was 0.85.

### MRI data acquisition

MRI images were acquired on a 3 T Siemens Prisma scanner with a 64-channel phase-array coil. Foam padding was used to minimize participant head movement and images were acquired along axial planes parallel to the anterior commissure-posterior commissure (AC-PC) line. A high-resolution magnetization prepared rapid gradient-echo (MPRAGE) sequence was used with the following parameters: 192 axial slices each 1.0 mm thick, skip = 0 mm, TR = 2,530 ms, TE = 2.98 ms, flip angle = 7°, FOV = 256 mm, matrix size = 448 × 512, voxel size = 0.5 × 0.5 × 1.0 mm. Each T1 image was visually inspected for excessive motion artifact and no participants were excluded based upon image quality.

### Voxel-Based Morphometry Analysis

VBM is a whole-brain processing method that can determine the neuroanatomical correlates of behavioral performance across participants through characterizing the differences in the volume of brain tissues (usually gray matter)^68^. In this study, VBM analysis was performed using Statistical Parametric Mapping (SPM8, Wellcome Trust Centre for Neuroimaging, University College London, UK) and theVBM8 toolbox (http://dbm.neuro.uni-jena.de/vbm) in MATLAB R2012b (Mathworks Inc., Sherborn, MA), following the suggested defaults of the VBM8 manual (http://dbm.neuro.unijena.de/vbm8/VBM8-Manual.pdf). First, T1-weighted images were segmented into gray matter (GM), white matter (WM) and cerebrospinal fluid (CSF) using the SPM standard tissue probability map^69^ with very light bias regularization (0.0001), 60 mm cutoff bias full-width half-maximum kernel (FWHM), affine regularization using the International Consortium for Brain Mapping (ICBM) space template – European brains, a warping regularization of 4, and a sampling distance of 3. The DARTEL algorithm (Diffeomorphic Anatomical Registration Through Exponentiated Lie Algebra)^70^ was used for spatial normalization for segmented gray matter, white matter, and CSF images, using the ‘IXI500_MNI152’ template, a spatial adaptive nonlocal means (SANLM) de-noising (multi-threaded) filter, a Markov Random Field (MRF) weighting of 0.15, and a light cleanup. The modulated normalized images (representing relative volume after correcting for brain size) were generated by correcting only for non-linear warping for the effect of spatial normalization, which represents the relative volume after correcting for different brain size (suggested by the VBM8 manual). The resulting modulated GM images were then smoothed with a 5-mm FWHM Gaussian kernel.

### Statistical Analysis

Statistical analyses of GMV data was performed using SPM8. Individual smoothed GMV images were entered into a voxel-wise generalized linear modeling (GLM) to identify regions whose rGMV was significantly associated with individual differences in trait modesty, controlling for age, gender and global GMV. An absolute threshold mask of 0.2 was used to avoid edge effects around the borders between GM and WM^71^. Global normalization was not performed. Statistical maps were thresholded at *p*_*uncorr*_ < 0.001 and p < 0.05 corrected for multiple comparisons at the cluster-level with family-wise error (FWE) correction.

Scatter plots were also created for each significant cluster for demonstrating purpose^40^. In which correlation coefficients were calculated using the trait modesty score and mean rGMV values of the clusters adjusted for age, gender and global GMV.
